# Biofilm presence and substrate type influence oyster larval settlement and survival

**DOI:** 10.1128/spectrum.00674-26

**Published:** 2026-10-07

**Authors:** Yuanqiu He, Xin Dang, Shiu C. Chung, Juan Diego Gaitan-Espitia

**Affiliations:** 1 The Swire Institute of Marine Science, School of Biological Sciences, The University of Hong Kong 25809, Hong Kong, China; 2 Lee Kum Kee Co., Ltd., Hong Kong, China; 3 Institute for Climate and Carbon Neutrality, The University of Hong Kong 25809, Hong Kong, China; Northwest A&F University College of Animal Science and Technology, Yangling, Shaanxi, China

**Keywords:** attachment, metamorphosis, larval selection, bacterial biofilm, substrate

## Abstract

Marine invertebrates with a bipartite life cycle typically undergo a developmental transition from pelagic larvae to benthic juveniles. The success of this transition largely depends on appropriate habitat and substrate selection, which is crucial for completing metamorphosis, subsequent development, and survival. While larvae are known to select microhabitats based on substrate-associated biofilms, whether the influence of these biofilms persists beyond post-metamorphosis remains poorly understood. Here, we investigated how microhabitat and biofilm types influence larval settlement choice and how these choices, in turn, affect settlement success and post-metamorphosis survival in the ecologically and economically important Hong Kong oyster (*Crassostrea hongkongensis*). The results indicate that biofilm presence positively influenced larval settlement, irrespective of substrate type. Following larval addition, biofilm profiles on substrates converged between control and treatment groups. In contrast, pre-existing biofilms had no significant effect on post-metamorphic survival, which instead depended primarily on substrate type. High-throughput sequencing of pre-existing biofilms on oyster shell substrates revealed six bacterial families enriched in association with higher larval settlement, including Cryomorphaceae, Rs-E47_termite_group, Bacteriovoracaceae, Rhodothermaceae, Vampirovibrionaceae, and Rubritaleaceae. Together, these findings suggest that substrate-associated biofilms primarily influence early settlement dynamics, whereas substrate type governs post-metamorphic survival. This decoupling highlights the distinct roles of microbial biofilm and substrate characteristics across different life-history stages, offering insights with potential implications for restoration and conservation strategies for marine invertebrates.

**IMPORTANCE:**

The transition from free-swimming larvae to bottom-dwelling juveniles represents a critical bottleneck for marine invertebrates, with direct implications for the recovery and resilience of coastal ecosystems. Using the Hong Kong oyster, an ecologically and economically important reef-building species, this study demonstrates that larvae preferentially settle on biofilm-covered shells and that this preference enhances settlement success. However, we show that the benefits of biofilm selection do not extend to post-metamorphic survival, which is instead governed by substrate type, revealing a previously unrecognized decoupling between the cues driving settlement and those determining subsequent survival. By characterizing the bacterial communities associated with higher larval settlement, this work identifies candidate microbial taxa that could inform the development of improved settlement substrates. Together, these findings provide a mechanistic foundation for optimizing restoration strategies for oyster reefs and offer a framework applicable to the conservation of other marine invertebrate species.

## INTRODUCTION

Marine invertebrates with a biphasic life history strategy are characterized by a critical developmental transition from pelagic larvae to benthic juvenile-adults (1, 2). This essential developmental phase (i.e., settlement and metamorphosis) is considered a key demographic bottleneck with marked changes in survival and subsequent development of post-settled larvae (3, 4). The process and success of larval settlement are particularly influenced by the surrounding environmental conditions (e.g., seawater pH, salinity, temperature, and food) and the characteristics of the available substrate (e.g., material, rugosity, and associated biofilm) (5, 6). When environmental conditions are adequate, larvae can employ diverse behavioral responses that enhance the likelihood of optimal habitat selection and settlement/metamorphosis success (7, 8). This capacity for microhabitat selection is hypothesized to be linked to substrate-associated biofilms (i.e., bacterial communities adhering to substrate surfaces [9]), their characteristics (e.g., structure, complexity, and age), and the nature of their chemical cues (i.e., soluble or surface-bound) (10). For example, in some ascidians (e.g., *Ciona intestinalis* and *Botrylloides violaceus*), swimming larvae tend to settle and survive at higher frequencies after selecting microhabitats with concrete substrata coated with young microbial biofilms (11). Building on this, it has been proposed that these and other types of man-made composite materials (i.e., cement, fiberglass, and plastics) can attract and promote larval settlement in a broad range of marine invertebrates (12). However, previous studies have suggested that the settlement process is also the result of a gregarious effect triggered by settled congeners (i.e., substratum-independent, group effect) (13). Under this perspective, larvae are more inclined to attach to substrates where their congeners gather, as this potentially enhances the likelihood of survival and development during the transition to a sessile lifestyle after metamorphosis (14). Cues signaling the presence of congeners (larvae and adults) can be potentially interpreted as an indicator of habitat suitability, promoting larval settlement independently of the background substratum (15). While previous studies have documented the ability of larvae to select microhabitats and substrate-associated biofilm components, it remains unclear whether the influence of existing biofilm has long-lasting effects that persist post-metamorphosis.

Marine invertebrate larvae use external chemoreceptors to navigate and detect environmental cues potentially indicating the optimal sites for settlement and metamorphosis (10). In addition to the chemical cues emitted by congeners, there are also signaling molecules (e.g., AHLs and TBP) produced by bacteria-dominated biofilms, which are suggested to be beneficial in promoting the settlement and metamorphosis of planktonic larvae (16). For instance, the marine bacteria *Pseudoalteromonas luteoviolacea* and *Cellulophaga lytica* produce protein complexes (tailocins) and lipopolysaccharide that can promote the metamorphosis of *Hydroides elegans* (17–19). Similarly, *Pseudoalteromonas* bacteria, isolated from coralline algae, produce a metamorphosis cue identified as tetrabromopyrrole, which induces changes in gene expression in the larvae of the coral *Acropora millepora* (16, 20). Considering the species-specific nature of these signaling cues, it is hypothesized that their effect on the settlement and metamorphosis of planktonic larvae would vary as a function of the diversity and structure of the bacterial biofilm community. While these principles apply broadly across marine invertebrates, their specific mechanisms and outcomes vary considerably among species, making species-specific investigations essential for understanding and managing settlement in ecologically and commercially important organisms. Here, we tested this hypothesis using the ecologically and economically important Hong Kong oyster, **Crassostrea** *hongkongensis*, assessing the influence of bacterial biofilms and substrate types on oyster larval microhabitat selection, settlement, and post-metamorphosis survival. Potential bacterial facilitators within the biofilm community were identified through 16S rRNA barcode sequencing. This study provides scientific evidence regarding the ability of marine invertebrate larvae to select congeneric substrata characterized by biofilm, which can enhance the success rate of larval settlement and post-metamorphosis survival. Here, we demonstrated the interplay between larval microhabitat selection and the role of microbial biofilms. This research enhances our understanding of how microbial communities influence larval settlement and metamorphosis in marine bivalves.

## MATERIALS AND METHODS

### Study system and larval developmental characteristics

Hatchery-produced 2-year-old oysters Crassostrea hongkongensis (also known as *Magallana hongkongensis*) were collected from Guandu, China (21.38°N, 110.40°E). The parental oysters were transported in moist conditions to an oyster hatchery (21.07°N, 110.27°E, Zhanjiang, Guangdong Province, China). Oysters used as broodstocks (~60 individuals) were washed and cleaned to remove biofouling epibionts, and they were maintained in three fiber-glass tanks (200 L each) and acclimated at pH_NBS_ 8.0, 15 ppt, and 28°C. Each culture tank contained ~20 individuals, which were fed three times a day with a combination of *Isochrysis* sp. and *Chaetoceros* sp., until gonads were fully matured. After 1 month, six females and three males with matured gonads were selected from each tank for stripped spawning. The fertilization and larval culture were conducted according to established procedures (4, 5). The suspended eggs and sperm were activated in seawater for 30 min. Motile sperm were added to the egg suspension at a ratio of 1:5 for artificial fertilization. Embryonic development was continuously observed under a microscope until the 16-cell stage. Fertilized embryos were then transferred into three 500 L black fiber-glass tanks at a final concentration of ~18 embryos per milliliter. At 14 days post-fertilization (when individuals reached the pediveliger stage with a visible foot and eyespot; size: ~300 µm), larvae were collected for the subsequent settlement bioassay experiment. Water conditions during larval culture and the experimental period are described in Table S1. Larvae were fed with a combination of *Isochrysis* sp. and *Chaetoceros* sp. (1 × 10^5^ cells/ml) throughout the experiment.

### Experimental design

**Fig 1:**
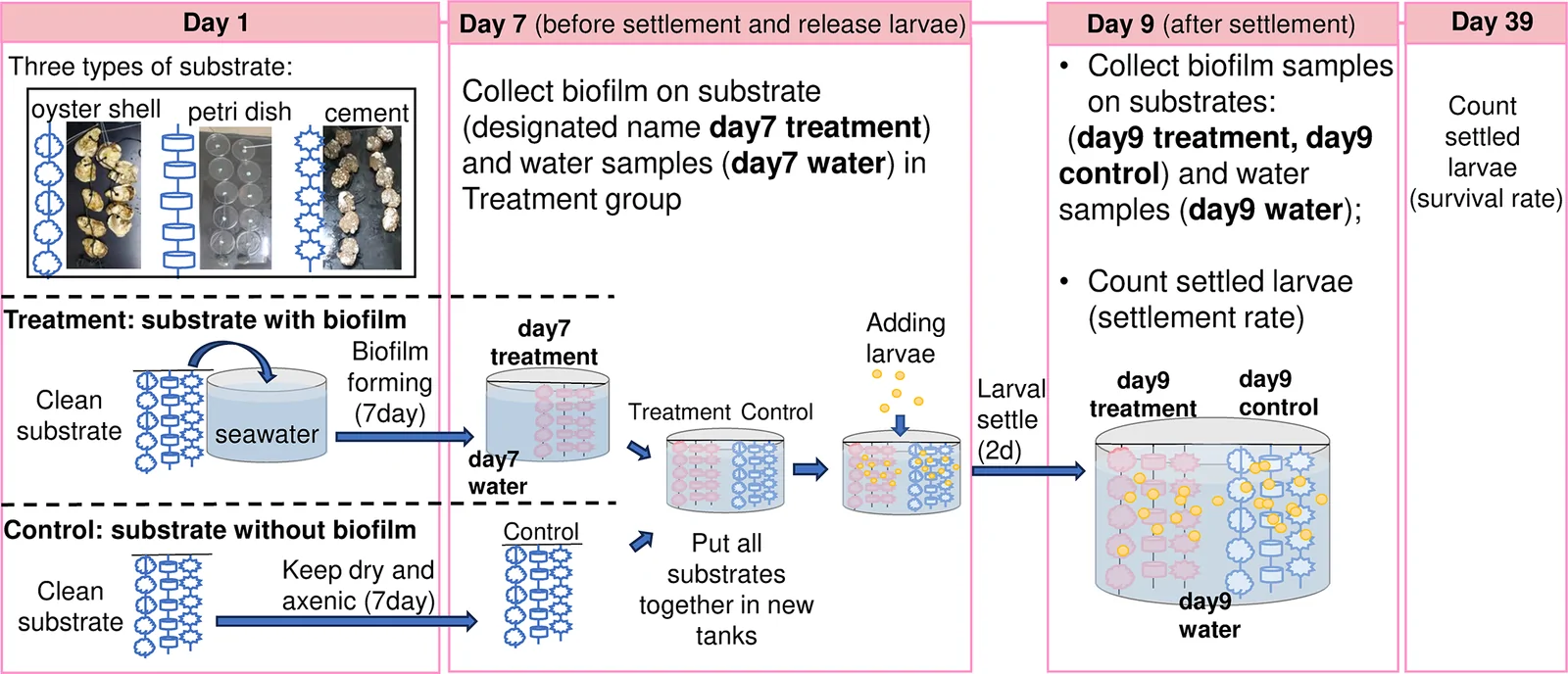
Overview of the biofilm-forming and larval settling experiment.

The pediveliger larvae require suitable substrates to settle before metamorphosis (21). As this process is critical for the recruitment and demographic dynamics of oyster populations, we assessed the influence of two variables (substrate type and biofilm) on larval settlement and survival. For the substrate type factor, oysters were offered three options that differed in chemical and physical properties (i.e., cement, plastic, and oyster shell). For the biofilm factor, we controlled the presence/absence of microbial communities attached to the substrates by immersing the different materials into natural seawater (i.e., allowing biofilm attachment) or by keeping them dry (i.e., preventing biofilm formation). Each substratum type was represented by five replicates attached together in a single string (Fig. 1). We used petri dishes (Falcon #1006, 50 × 9 mm) for the plastic substrate, oyster shells for the natural organic/inorganic substrate, and a mix of cement, sand, and small rocks for the artificial inorganic substrate. All tanks and substrate units were cleaned and washed with 10% bleaching solution and dried at room temperature before use.

### Biofilm formation

The three types of substrates used for the attachment of oyster larvae were immersed for 7 days in a plastic tank (20 L) filled with non-filtered, natural seawater to allow biofilm formation (Fig. 1). A total of three tanks were used as independent replicates for biofilm formation. The experimental substrates with formed biofilms were collected on the 7th day (one replicate per tank). The 7-day immersion period was set to allow natural biofilm development according to the common hatchery practices and previous studies (22, 23). The average temperature of seawater during the immersion period was 30.4°C. We randomly selected one piece from each substrate type (oyster shell/cement/plastic) from each tank (three replicates per substrate) and sampled the surface with sterilized cotton swabs. These samples represented the “Before Settlement” biofilms (designated group day7-treatment, abbreviated to d7t). In parallel, water samples were collected by filtering 1 L of water from each tank replicate through 0.22 μm membrane filters (Millipore). These samples were assigned as the “Before Settlement Water” microbiomes (designated group day7-water, abbreviated as d7w). Immediately after collection, all samples were rapidly stored at −80°C for subsequent analysis.

### Settlement selection assay

Six tanks were filled with 20 L sand-filtered natural seawater and aerated during the experimental period (from day 7 to day 9, the average temperature of the seawater was 29.01°C, Table S1). In each tank, six strings made from different substrates (two strings per substrate type) were placed together for larval settlement. Half of the strings contained 7-day-old biofilms, while the other half were without biofilm. Each string consisted of five substrate replicates connected by the same rope (Fig. 1). Approximately 10,000 pediveliger oysters (individuals with an eye-spot and a foot, ~300 µm) were added to each tank using a beaker for thorough mixing. Two liters of algae (*Chaetoceros* sp. at 1 × 10^5^ cells/ml) were added to the tanks 1 hour later to ensure larval survival and promote settlement. The number of settled larvae on each substrate (both sides) was collected with magnifying glasses after 48 h within a 50 mm round area on each type of substrate. Sterilized cotton swabs were used to collect the surface biofilm on the substrates as previously described. Samples were collected from substrates with biofilms (designated group day9-treatment, abbreviated to d9t), no biofilm substrates (group day9-control, d9c), and the water group (day9-water, d9w). The surviving larvae attached to the substrates were counted on day 39 using the same method described for day 9. The average temperature of seawater during the 30-day culture is 30.46°C.

### DNA extraction, amplification, sequencing, and quality control

Biofilm samples from oyster shell substrate were used to extract DNA for 16S rDNA amplicon sequencing. Total DNA from swabs and water samples (collected from three of the six tanks) was extracted and used for subsequent 16S rRNA gene amplification sequencing to identify the composition of the biofilm community. Total DNA was extracted using the TGuide S96 Magnetic Soil/Stool DNA Kit (TIANGEN BIOTECH [BEIJING] CO., LTD) according to the manufacturer’s instructions. A Qiagen Powerlyzer and a water bath were used to release bacterial cells from the cotton swabs. The quality of the extracted DNA was examined using a Synergy HTX Multi-Mode Reader (Agilent), and the amplified products were checked with 1.8% agarose gel electrophoresis. The 16S rRNA gene amplicon sequencing analysis was conducted by using an Illumina NovaSeq 6000 with the primers 338F (5′- ACTCCTACGGGAGGCAGCA-3′) and 806R (5′- GGACTACHVGGGTWTCTAAT-3′) at Biomarker Technologies Co., Ltd. (Beijing, China). Raw reads were filtered, and barcodes were removed using Trimmomatic v0.33. QIIME2 (version 2022.2) was used for removing primer sequences, denoising, and assembling paired-end sequences (DADA2). The obtained amplicon sequence variants (ASVs) were taxonomically assigned with the Naive Bayes classifier using the Silva reference database (classifier_silva_13_8_99). Raw reads were deposited in the NCBI database with accession number PRJNA1146188.

### *In silico* analysis

The microbial feature table generated from QIIME2 was imported into R for further analysis. The package vegan (version 2.6.4) was used to analyze alpha- and beta-diversity (method = bray) with the rarefied feature table. Differential abundance analysis was performed in R using the ANCOMBC (version 1.4.0) and ALDEx2 (version 1.26.0), and the ASVs that existed in only one sample were filtered out. The ANCOMBC analysis was conducted comparing group day7-treatment, day9-treatment, and day9-control (with the latter set as the reference group). The ALDEx2 analysis was performed for paired samples from groups day7-treatment and day9-treatment (mc.samples = 128). The families that changed significantly in the ANCOMBC analysis were selected for visualization using the package pheatmap (version 1.0.12), based on their relative abundance. The results from the ALDEx2 paired comparison were visualized using the package microbiomeutilities (version 1.00.17). The Phylogenetic Investigation of Communities by Reconstruction of Unobserved States (PICRUSt) analysis was performed to predict the functions from the microbial profile using PICRUSt2 (version 2.4.1). Differences in the functional abundances of pathways were tested using ANCOMBC from the output of PICRUSt2, with the day9-control group designated as the reference group.

### Statistical analyses

The counts of settled larvae within a standardized area on each substrate were analyzed with a generalized linear mixed model with the package lme4 (version 1.1.32) in R (version 4.1.3). A negative-binomial mixed model (estimated using ML and Nelder-Mead optimizer) was fitted to assess the influence of substrates and biofilm on settlement success (formula: count ~ substrates + biofilm + 1|Tank ID). Survival data collected after 1 month were analyzed using a logistic mixed model with binomial error distribution, also implemented in lme4 (formula: survive ~ substrates + biofilm + 1|Tank ID). Both models included Tank ID as a random effect to account for variation among the six settlement tanks. To test for potential non-independence among substrates originating from the three biofilm-formation tanks, an additional full model was fitted that included biofilm-formation tank as a nested random effect [formula: count ~ 1 + (1|biofilm tank/settlement tank)]. This model indicated that the biofilm-formation tank did not introduce non-independence among settlement tanks (detailed results are shown in Fig. S1). Model assumptions were checked with the R package Performance (version 0.10.2). Tukey’s multiple comparisons were used to test for differences in the means of the Shannon index in R. For beta diversity, a PERMANOVA test was performed to assess significant differences among groups with 999 permutations based on Bray-Curtis distance.

## RESULTS

### The effect of biofilm and substrate on the number of larval settlement and survival

**Fig 2:**
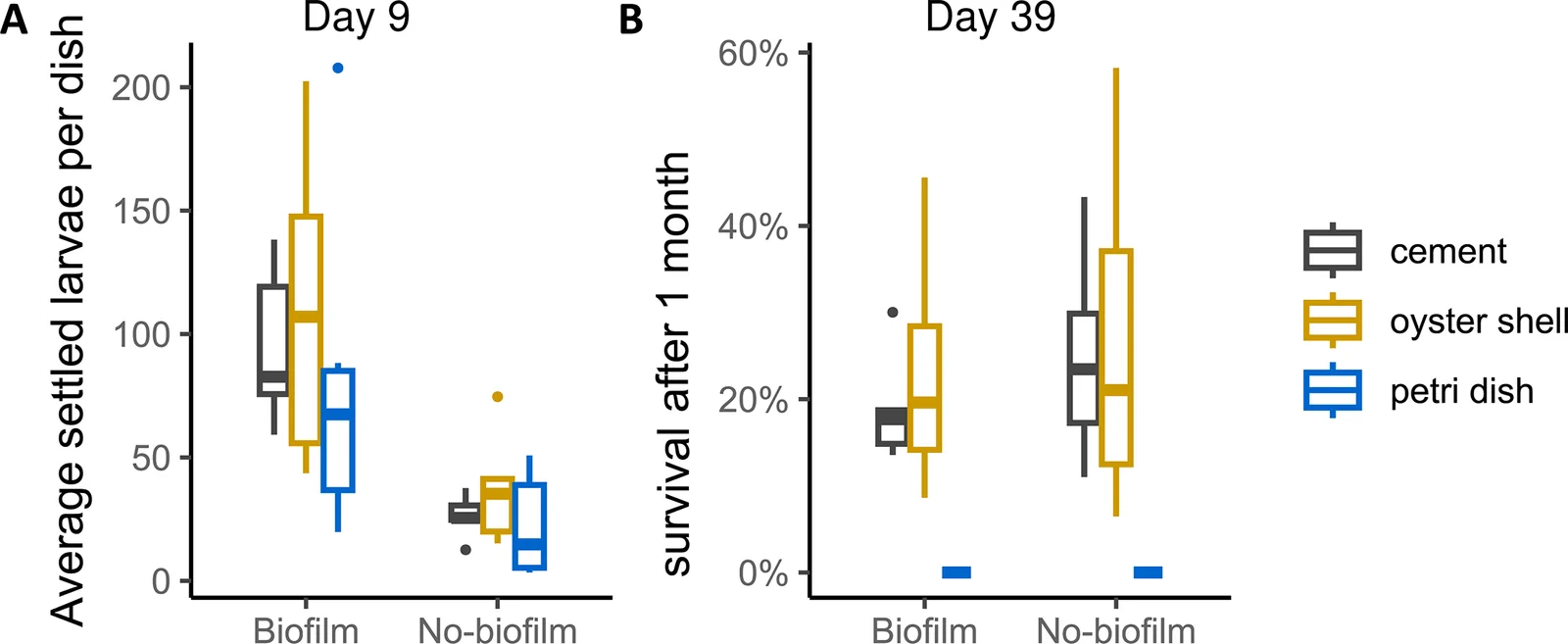
Settled larvae count and survival percentage among different treatments on day 9 and day 39. (**A)** The average number of settled larvae on different substrates with or without biofilm on day 9. (**B)** The survival rate of larvae among treatments on day 39. Bars represent 1.5 × IQR (inter-quartile range) from the hinge.

The negative-binomial mixed model revealed a significant positive effect of biofilm on larval settlement (*P* < 0.001). Compared to the plastic substrate (petri dish) with biofilm, oyster shell substrates significantly increased the number of settled larvae (*P* = 0.006), while settlement did not differ significantly between cement and plastic substrates (*P* = 0.205; Table 1). No significant interaction was detected between the substrate type and the biofilm treatment (Fig. 2A). After 1 month, post-metamorphic survival differed significantly among substrate types (Fig. 2B). Plastic substrates exhibited markedly lower survival rates, while oyster shell and cement substrates showed comparable survival (~20%), regardless of biofilm treatment (substrate: F₁,₂ = 19.776, *P* < 0.001; biofilm: F₁,₁ = 0.30, *P* = 0.585).

**TABLE 1:** Summary of model results testing the effect of substrate and biofilm on larval settlement on day 9 and day 39^*a*^

Coefficient	Estimate	Std. error	*Z* value	Pr(>|*z*|)
Day 9 (negative binomial mixed model)				
(Intercept)	4.287	0.16	26.77	<0.0001***
Substrate cement	0.211	0.166	1.268	0.205
Substrate oyster shell	0.479	0.174	2.758	0.006 **
No biofilm	−1.223	0.131	−9.356	<0.0001 ***
Day 39 (binomial mixed model)				
(Intercept)	−1.80803	0.12166	−14.861	<2e−16 ***
Substrate oyster shell	0.09658	0.06669	1.448	0.148
Substrate petri dish	−6.06945	0.99441	−6.104	1.04e−09***
No biofilm	0.04102	0.07513	0.546	0.585
Random effect	Variance	Std.dev.		
Day 9 (negative binomial mixed model)				
Tank ID	0.04171	0.2042		
Day 39 (binomial mixed model)				
Tank ID	0.07285	0.2699		

^*a*^ Significant codes: ***, *P* < 0.001; **, *P* < 0.01; *, *P* < 0.05. Degrees of freedom: day 9 model: 172; day 39 model: 10,914 degrees of freedom).

### Biofilm formation and community diversity

**Fig 3:**
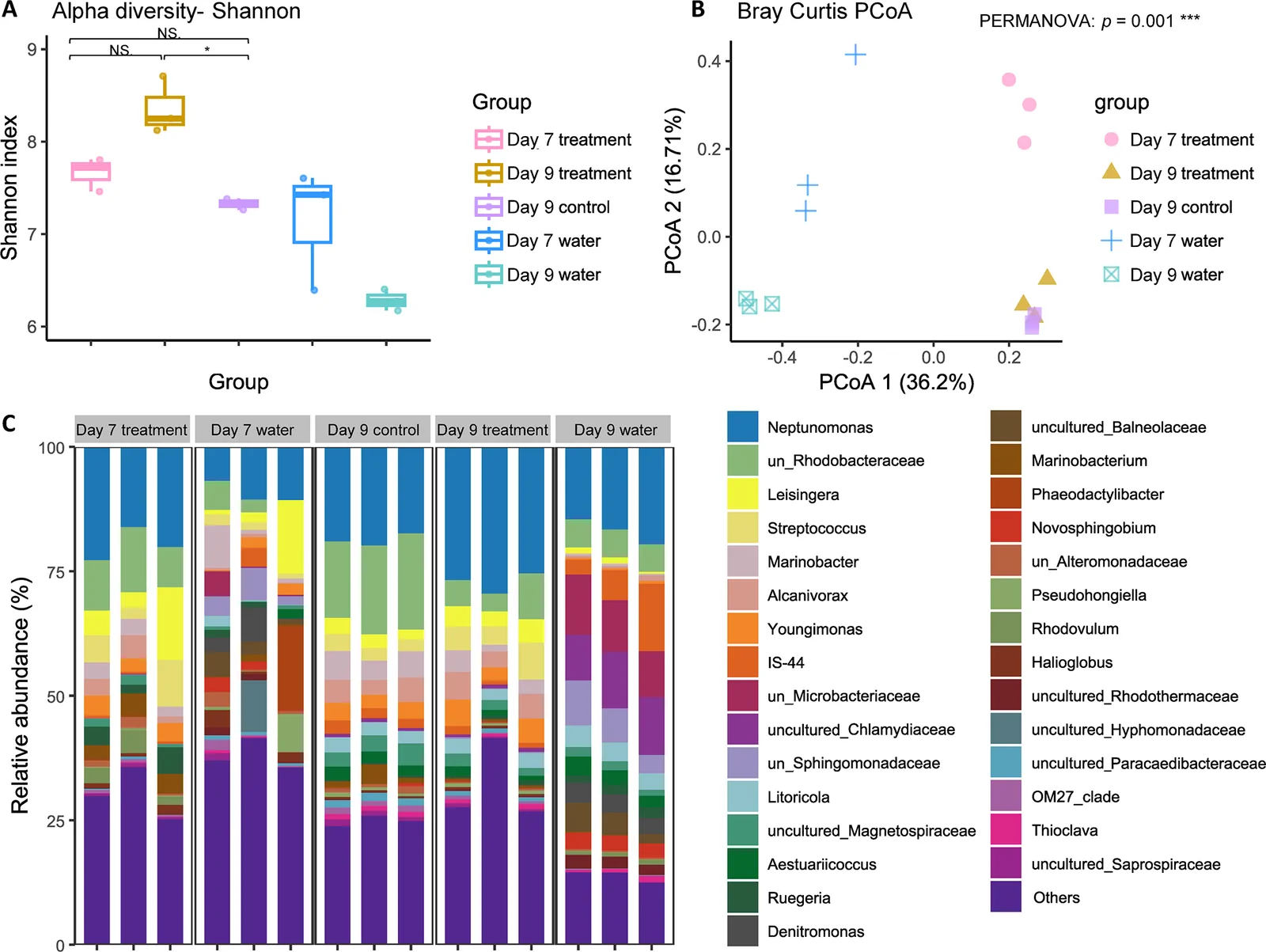
The bacterial diversity and community profile among groups. (**A**) Alpha diversity of biofilm on oyster shell substrate. Bars represent 1.5 × IQR [inter-quartile range] from the hinge. (**B**) Beta-diversity on oyster shell substrate. (**C**) The bacterial composition among groups at the genus level, with three replicates in each group.

The presence of biofilm significantly increased the number of settled larvae on all substrates, and oyster shell attracted the greatest number of settled larvae (Fig. 2A). Therefore, we focused only on sequencing of the biofilm samples from oyster shells to examine the differences between biofilm and no-biofilm substrates. The 16S rRNA gene amplicon sequencing generated 1,045,332 high-quality sequences, which were assigned to 10,752 ASVs. The oyster shells with biofilm post-settlement (day9-treatment) exhibited the highest Shannon index (*P* = 0.026) among all groups (Fig. 3A). The day7-treatment had the second-highest Shannon index, indicating that the 7-day immersion period enriched the microbial community attached to the substrate. Together, these results suggest that the highest diversity observed in the day9-treatment group was built upon the pre-existing biofilm community established during the day7-treatment period.

Overall microbial composition differed significantly among all groups (Fig. 3B: *R*^2^ = 0.6865, *F* = 5.4758, *P* = 0.001). However, principal coordinate analysis (PCoA) revealed that the microbial communities on substrates with and without biofilms (day9-treatment and day9-control) were not clearly distinct (Fig. 3B). In contrast, the biofilm community that developed during the 7-day immersion period (day7-treatment) clearly diverged from the day9-treatment group along the PCoA2 axis. Together, the Shannon index and the PCoA results indicate similar microbial community structures between the day9-treatment and day9-control groups, as evidenced by the consistent clustering. Furthermore, the surface-associated bacterial communities in the day7-treatment group resembled those of the surrounding water (day7-water) (Fig. 3C). Following larval addition on day 9, however, the bacterial communities on oyster shell substrates converged between the control and treatment groups, becoming increasingly similar to one another. In contrast, the water samples’ microbiome shifted over time, with the day9-water community differing notably from that of day7-water.

### Differential abundance analysis

**Fig 4:**
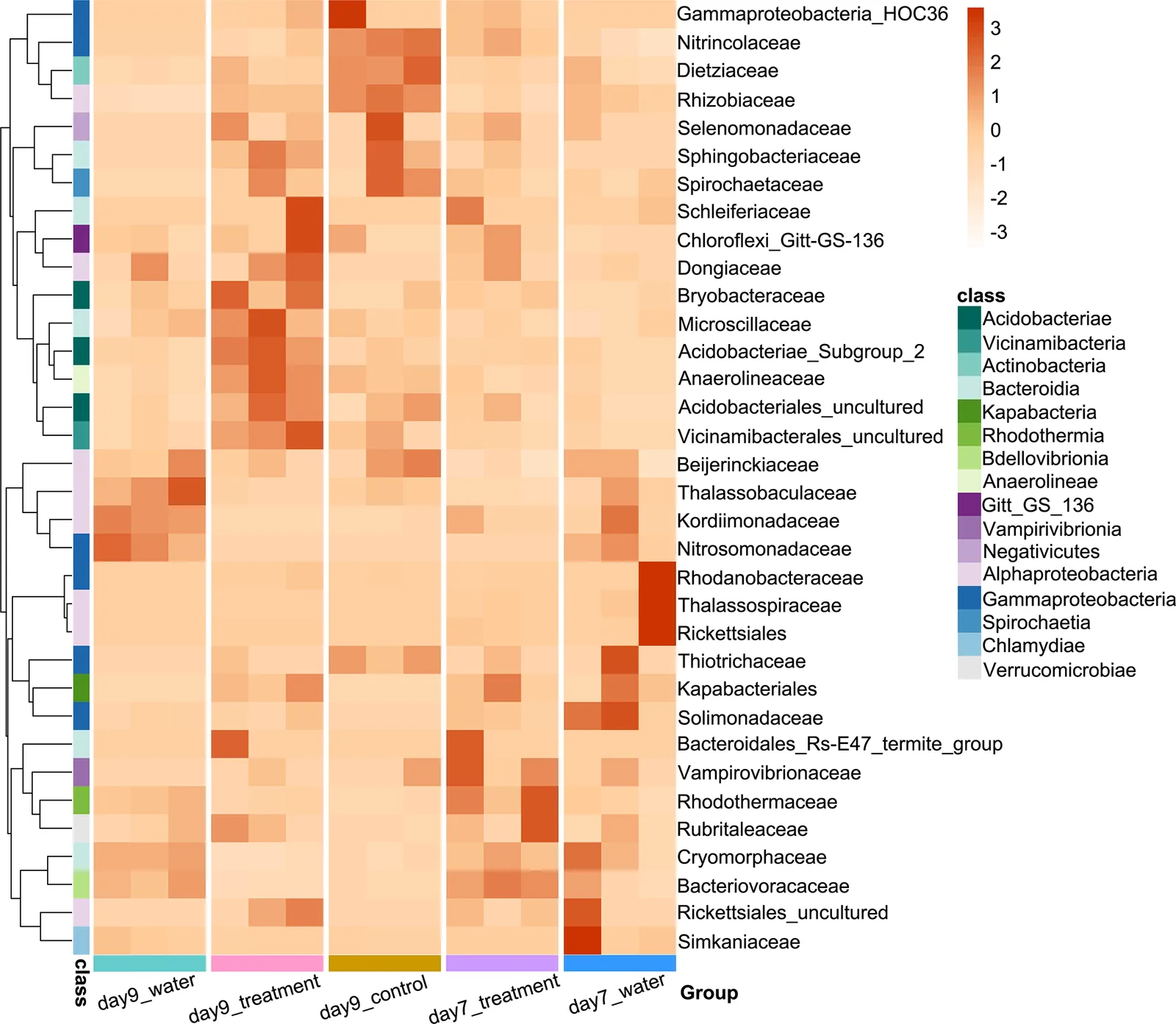
The heatmap of 34 differential families from ANCOM-BC analysis. Water samples from day 7 and day 9 were added as reference. Values represented the relative abundance of family that scaled by row.

The differential abundance analysis (ANCOM-BC) across the three groups (i.e., day7-treatment, day9-treatment, and day9-control) identified 34 families with significant differences in relative abundance. Prior to larval addition, the 7-day immersion period resulted in biofilms enriched in the families Cryomorphaceae, Rs-E47_termite_group, Bacteriovoracaceae, Rhodothermaceae, Vampirovibrionaceae, and Rubritaleaceae (Fig. 4). Following larval addition, the biofilm communities on substrates from the treatment and control groups each harbored distinct sets of dominant families. Relative to the control group, 24 families showed significantly higher abundance in the treatment group (positive beta value, *P* < 0.05), whereas 7 families showed significantly reduced abundance. The families Schleiferiaceae, Chloroflexi_Gitt_GS_136, Dongiaceae, Bryobacteraceae, Microscillaceae, Acidobacteriae_Subgroup_2, Anaerolineaceae, Acidobacteriales_uncultured, and Vicinamibacterales_uncultured showed higher relative abundance in the treatment groups, while the substrates from the control group showed enrichment in the families Gammaproteobacteria_HOC36, Nitrincolaceae, Dietziaceae, Rhizobiaceae, Selenomonadaceae, Sphingobacteriaceae, and Spirochaetaceae. Additionally, three families (Cryomorphaceae, Bacteriovoracaceae, and Thalassospiraceae) showed significantly increased abundance prior to larval addition but declined following larval settlement (Table S2).

PICRUSt-based functional prediction identified 26 putative pathways that were significantly enriched in the treatment groups compared to the control group (Table S3). Five pathways were enriched in both treatment groups, including nitrifier denitrification, sitosterol degradation to androstenedione, D-cycloserine biosynthesis, superpathway of mycolyl-arabinogalactan-peptidoglycan complex biosynthesis, and benzoyl-CoA degradation II. The top five pathways enriched in the control group included mycolyl-arabinogalactan-peptidoglycan complex biosynthesis, 1,5-anhydrofructose degradation, 4-hydroxyphenylacetate degradation, mevalonate pathway I, and the superpathway of geranylgeranyldiphosphate biosynthesis I (via mevalonate).

**Fig 5:**
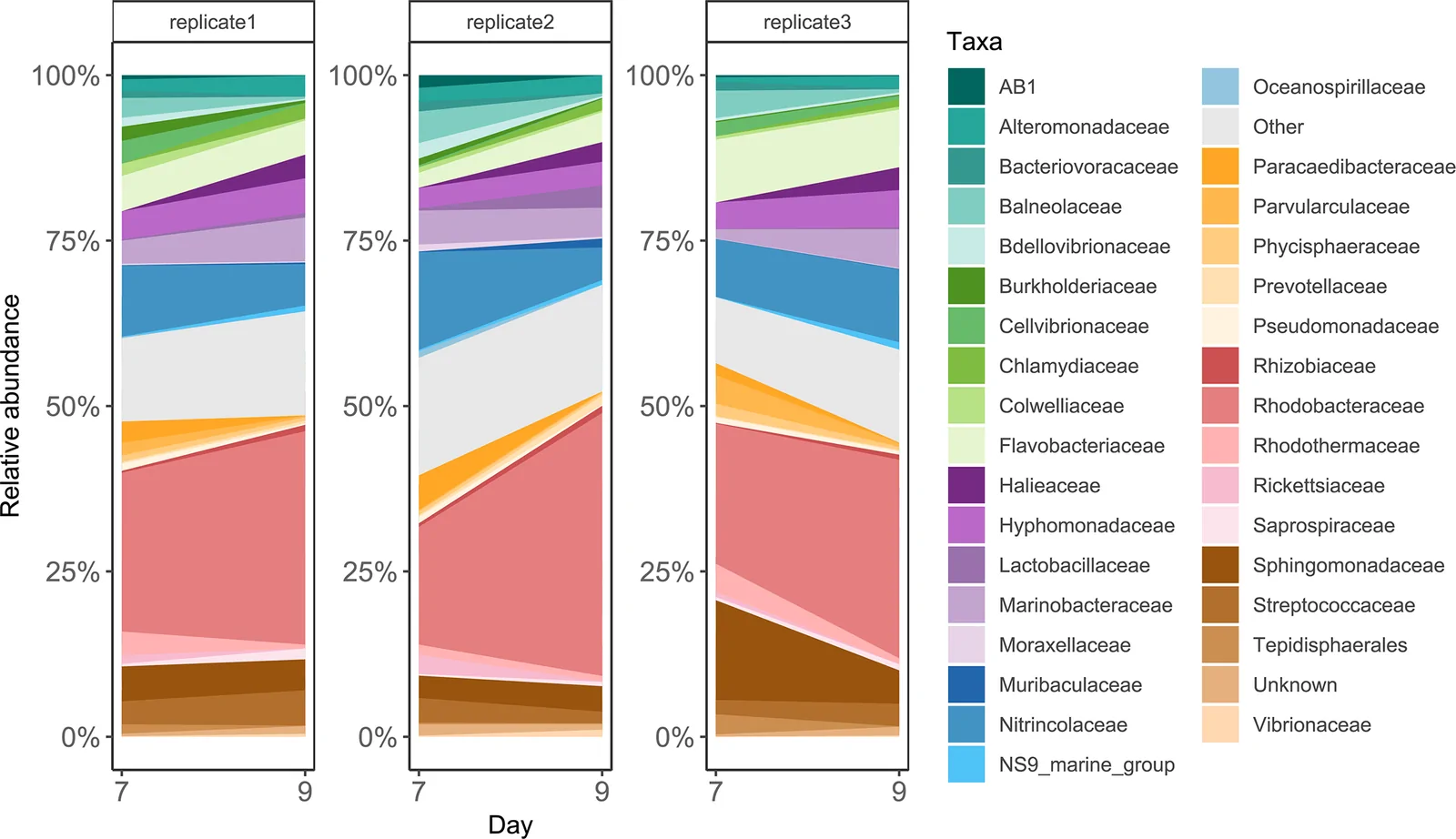
The microbial changes at the family level in each replicate over time. Day 7 represents the substrates that completed a 7-day immersion before adding larvae. Day 9 represents the substrate 48 h after adding larvae. Each treatment has three replicates.

Upon larval settlement, the overall biofilm composition did not vary significantly at the family level, as determined by the ALDEx2 paired analysis. However, the relative abundances of several families changed, particularly among the 11 dominant groups (those with a relative abundance above 1%) (Fig. 5). The families Halieaceae, Chlamydiaceae, Alteromonadaceae, Rhizobiaceae, NS9_marine_group, and Vibrionaceae were significantly enriched in the substrate biofilm after the larval settlement (ALDEx2 analysis, *P* < 0.05). Conversely, a significant decrease in relative abundance was observed in the families Balneolaceae, Bacteriovoracaceae, Paracaedibacteraceae, Rickettsiaceae, and Pseudomonadaceae, following larval settlement (Table S4: *P* < 0.05).

## DISCUSSION

### Biofilms promote larval settlement, whereas substrate type influences post-metamorphic survival

Larval settlement is a pivotal developmental event that determines both individual survival and the demographic trajectory of oyster populations. This study demonstrates that biofilm presence and substrate type influence different stages of the larval-to-juvenile transition: biofilm presence significantly increased larval settlement, whereas post-metamorphic survival was associated with substrate type. Notably, our results revealed that natural biofilms formed in the seawater can enhance settlement rates across all substrate types tested, a pattern consistent with previous findings in another oyster species, *Crassostrea virginica* (24).

Despite the overall positive effects of biofilm presence observed across all substrate types, significantly fewer larvae settled on plastic substrates (petri dish) compared with oyster shells (Fig. 2A). This difference is likely attributable to the physical properties of the substrate surface. Previous studies have reported that larvae of the pearl oyster, *Pinctada martensii,* preferentially settle on rough substrates (25). Because oyster larvae (*Crassostrea gigas*) secrete bioadhesive substances from the foot to facilitate attachment during substrate selection (26), we infer that rougher surfaces may facilitate the adhesion of these secretions, thereby promoting settlement. This interpretation is supported by findings from oyster reef restoration studies, in which larvae preferentially settled in high-roughness microhabitats characterized by lower shear stress, conditions that favor attachment stability (27, 28). In addition to these surface-related physical properties, differential settlement responses may also reflect a gregarious response, whereby biogenic substrates (in particular, oyster shell as a congeneric substrate) attract more larvae and enhance settlement relative to non-biogenic substrates. This gregarious behavior has been previously documented in other oyster species, including *C. virginica* and *Pinctada fucata* (29–31), and may promote the rapid establishment of wild populations by providing conspecific cues associated with secure and favorable habitats. Nevertheless, because the current experimental design did not include a control using congeneric adult chemical extracts in the absence of biofilm, we cannot disentangle this potential gregarious effect from the biofilm effect observed here. Additional studies are therefore needed to clarify the underlying mechanisms and evaluate the generality of this phenomenon.

Intriguingly, while biofilm presence significantly increased the settlement rate across different substrate types, it did not improve the survival or development of larvae post-settlement. This pattern suggests that the factors promoting larval settlement may differ from those that determine post-metamorphosis survival. In other words, biofilms may function primarily as early settlement cues, possibly through their surface physical properties and/or associated biochemical substances, without necessarily providing longer-term benefits for juvenile performance. A similar result was reported in sea urchin larvae, where higher settlement was observed in biofilm treatments but equivalent survival was recorded after 25 days (32). Our results therefore indicate that while biofilm presence can influence the decision to settle, post-settlement survival may depend more strongly on other environmental factors, particularly substrate characteristics. This distinction highlights the importance of evaluating both settlement and survival when assessing habitat quality, as conditions that attract larvae do not always confer advantages for post-metamorphic survival.

All settled larvae on petri dishes died after 1 month, regardless of biofilm presence. This finding suggests that an unsuitable substrate choice can directly compromise larval survival and growth after metamorphosis. Plastic substrates may release toxic leachates that increase larval mortality (33). Furthermore, their smooth surface likely provides fewer microhabitats for newly settled spat, further increasing mortality risk (29). Our findings highlight a potential limitation of settlement studies that rely solely on endpoints measured at the conclusion of the settling process (usually ~48 h), as such approaches may fail to capture post-attachment survival and growth, and therefore cannot reliably determine whether a given substrate is ultimately beneficial or detrimental to post-metamorphic success. We recommend that future substrate-selection studies place greater emphasis on the long-term survival and growth of post-metamorphosis larvae, rather than relying exclusively on short-term settlement metrics.

### Enriched bacterial families and functional pathways in substrate biofilms

Bacterial diversity in the treatment group was highest following larval addition, reflecting the combined contributions of taxa from both the 7-day immersion period and the larval culture. Beta diversity analysis indicated that, regardless of pre-existing biofilm presence, microbial community structure on substrate surfaces converged after 48 h of larval addition. This convergence suggests that the introduction of larvae and algae solution reduced the initial compositional differences between biofilm and non-biofilm treatments. Such an effect implies that the microbes associated with oyster larvae and algae exerted a strong influence on substrate biofilm composition, resulting in the overlap observed between treatment and control groups in the PCoA analysis. The rapid shift in bacterial community after larval addition in treatment groups may be attributable to the large quantities of larvae, algae, and their associated microbiota colonizing the substrate surfaces, thereby substantially altering the biofilm composition in both groups. Consequently, the pre-existing biofilm established during the 7-day immersion period did not appear to determine the final microbial structure of the substrate biofilm after larval addition. Previous studies have demonstrated that extracellular polymeric substances (EPS) within biofilms can enhance the larval settlement on substrates (34, 35). It is therefore possible that the higher settlement rates observed with pre-existing biofilms were mediated, at least in part, by the EPS present on the substrate surface prior to larval introduction.

Seven-day immersion of substrates in seawater resulted in the formation of biofilms characterized by six enriched bacterial families. Cryomorphaceae is widely distributed in mildly saline and marine environments, although its ecological functions remain poorly understood (36). The Rs-E47_termite_group and Rubritaleaceae are symbiotic bacteria that may confer benefits to host health (37, 38). Rhodothermaceae has been reported to exhibit cellulolytic and xylanolytic activities (39). Vampirovibrionaceae are obligate predators of algae, such as Chlorella (green algae), causing host cell lysis (40). Bacteriovoracaceae are obligate predators of gram-negative bacteria and have the potential to inhibit pathogenic bacteria (41). Although the functional roles of these families in larval settlement remain poorly understood and no specific chemical settlement cues have yet been identified from these taxa, their enrichment in pre-existing oyster shell biofilms, which coincided with higher larval settlement, suggests that they may represent candidate taxa associated with settlement outcomes. However, these associations remain correlative, and their functional significance has yet to be experimentally validated. We therefore infer that larval settlement is primarily driven by a physical response to the presence of a mature biofilm layer, while certain enriched bacterial families may provide ancillary benefits to settling larvae through mechanisms that remain to be elucidated.

Seven bacterial families were found in higher abundance in the control group compared to the biofilm treatment groups. The most significantly enriched families in the control were Nitrincolaceae, Rhizobiaceae, Dietziaceae, and Beijerinckiaceae. Nitrincolaceae is commonly found in marine and saline lake environments (42), while Rhizobiaceae are nitrogen-fixing symbionts (43). Dietziaceae has been characterized as an opportunistic pathogen (44), and Beijerinckiaceae are generalist organotrophs (45) known to dominate the gut bacterial communities of marine fish (46). The broad distribution of these families in marine environments may explain their enrichment in the control substrate biofilm. As key determinants of biofilm adhesive properties (10), their presence may have contributed to facilitating oyster larval attachment.

The enriched pathways predicted by PICRUSt suggest that the biofilms were at different developmental stages. The treatment groups on day 7 and day 9 showed enrichment of pathways associated with more established biofilm communities, indicating that these biofilms had likely progressed beyond the initial rapid-growth stage and entered a more mature phase (9). Such mature biofilms may provide a more stable microbial environment on the substrate surface, which could be beneficial for larval settlement. For example, D-cycloserine biosynthesis can produce antibiotics that may inhibit competing microorganisms (47). Enrichment of the superpathway of mycolyl-arabinogalactan-peptidoglycan complex biosynthesis contributes to a more stable cell wall structure and reduced permeability to environmental solutes (48). Additionally, higher predicted abundance of nitrifier-denitrification pathways in mature biofilms may reflect oxygen-limited microniches, where redox gradients support closely coupled ammonia oxidation and nitrifier denitrification (49). In contrast, the control group biofilm, having been immersed in seawater for only 2 days, likely represented an earlier developmental stage. Accordingly, the enriched pathways in the control group were predominantly associated with biofilm formation rather than maturation. The superpathway of polyamine biosynthesis I and the mevalonate pathway I have both been reported to promote biofilm formation (50, 51), while other enriched pathways appear to support supplementary carbon energy acquisition (52). Compared with the more established biofilms in the treatment groups, these early-stage biofilms may have been less structurally stable and therefore less effective in providing a favorable microbial habitat for settling larvae. Overall, these predicted functional differences suggest that the biofilm developmental stage may indirectly affect larval settlement outcomes. Future research directions could include (i) isolation of specific biofilm-associated microbial consortia and functional validation of their roles in oyster larval settlement and survival; (ii) metabolite analysis of the biofilm-associated substances to help elucidate the underlying chemical mechanisms; and (iii) the application of microscopic methods to monitor the physical and behavioral interactions of larvae with biofilms.

### Conclusion

In this study, we conducted a biochoice assay using Hong Kong oyster larvae to evaluate their substrate selection behavior across three different substrate types in the presence and absence of biofilm. Our results confirmed the pivotal role of biofilm in promoting larval settlement, irrespective of the substrate type. Moreover, oyster shells consistently attracted the highest number of settling larvae among the tested substrate materials. While post-metamorphic survival rates did not differ between oyster shell and cement substrates, both markedly outperformed plastic substrates, on which no larvae survived beyond 1 month. These results indicate that factors enhancing larval settlement do not necessarily align with those promoting post-metamorphic survival, emphasizing the importance of evaluating both stages when selecting materials for reef restoration. Taken together, these findings highlight oyster shell as a promising substrate for oyster reef restoration, given its capacity to promote both larval settlement and post-metamorphic survival. For example, oyster shells may represent a suitable substrate material for oyster reef restoration. Our 16S rRNA gene sequencing results further suggest that developmental shifts in oyster shell-associated biofilms may be linked to larval settlement responses, pointing to a potential microbial dimension underlying substrate suitability. Nevertheless, since microbial community analyses were limited to oyster shell substrates in this study, further work comparing biofilm communities across a broader range of restoration materials is needed to assess the generality of these patterns.

## Data Availability

The 16S rRNA gene amplification sequencing data reported in this study have been deposited to the NCBI Sequence Read Archive (SRA) with accession number PRJNA1146188. Water quality data and larval settlement data are available in DOI: http://doi.org/10.6084/m9.figshare.28853459.

## SUPPLEMENTAL MATERIAL

Fig. S1 spectrum.00674-26-s0001.docx The summary of pseudo-replication test.

Supplemental tables spectrum.00674-26-s0002.xlsx Tables S1 to S4.

ASM does not own the copyrights to Supplemental Material that may be linked to, or accessed through, an article. The authors have granted ASM a non-exclusive, world-wide license to publish the Supplemental Material files. Please contact the corresponding author directly for reuse.
