# Supplementary material for: Biofilm presence and substrate type influence oyster larval settlement and survival
Source: Microbiol Spectr. 2026 Oct 7;14(11):e00674-26. doi: 10.1128/spectrum.00674-26 (PMC13729213; doi:10.1128/spectrum.00674-26)
Supplement: Supplementary file 1 [file spectrum.00674-26-s0001.docx]

**A**

**B**

**Figure S1. The summary of pseudoreplication test**.

(Note: A: Larvae count in each tank from different substrate, no obvious pattern between the tanks from same initial bucket. B: we performed a likelihood ratio test using mixed‑effects models implemented in the lme4 package. For each of the three material types (cement, Shell, and petri dish), we compared a null model including only tank as a random intercept (number ~ 1 + (1|tank)) against a full model that additionally included bucket as a nested random effect (number ~ 1 + (1|bucket/tank)). The results consistently showed no improvement in model fit when the bucket effect was added: for all three types, the likelihood ratio test gave Chisq = 0, df = 1, p = 1. The variance component for the bucket random effect was estimated to be zero in each case. These findings indicate that there is no significant systematic variation attributable to the culture buckets; the variability between two tanks within the same bucket is statistically indistinguishable from that between tanks from different buckets.)
